# Morphology and Phylogeny of a New Species of Anaerobic Ciliate, *Trimyema finlayi* n. sp., with Endosymbiotic Methanogens

**DOI:** 10.3389/fmicb.2018.00140

**Published:** 2018-02-19

**Authors:** William H. Lewis, Kacper M. Sendra, T. Martin Embley, Genoveva F. Esteban

**Affiliations:** ^1^Institute for Cell and Molecular Biosciences, Newcastle University, Newcastle upon Tyne, United Kingdom; ^2^Bournemouth University, Faculty of Science and Technology, Department of Life & Environmental Sciences, Poole, United Kingdom

**Keywords:** anaerobic, ciliate, endosymbiont, methanogen, *Trimyema*, phylogeny, *Methanocorpusculum*

## Abstract

Many anaerobic ciliated protozoa contain organelles of mitochondrial ancestry called hydrogenosomes. These organelles generate molecular hydrogen that is consumed by methanogenic Archaea, living in endosymbiosis within many of these ciliates. Here we describe a new species of anaerobic ciliate, *Trimyema finlayi* n. sp., by using silver impregnation and microscopy to conduct a detailed morphometric analysis. Comparisons with previously published morphological data for this species, as well as the closely related species, *Trimyema compressum*, demonstrated that despite them being similar, both the mean cell size and the mean number of somatic kineties are lower for *T. finlayi* than for *T. compressum*, which suggests that they are distinct species. This was also supported by analysis of the 18S rRNA genes from these ciliates, the sequences of which are 97.5% identical (6 substitutions, 1479 compared bases), and in phylogenetic analyses these sequences grouped with other 18S rRNA genes sequenced from previous isolates of the same respective species. Together these data provide strong evidence that *T. finlayi* is a novel species of *Trimyema*, within the class Plagiopylea. Various microscopic techniques demonstrated that *T. finlayi* n. sp. contains polymorphic endosymbiotic methanogens, and analysis of the endosymbionts’ 16S rRNA gene showed that they belong to the genus *Methanocorpusculum*, which was confirmed using fluorescence *in situ* hybridization with specific probes. Despite the degree of similarity and close relationship between these ciliates, *T. compressum* contains endosymbiotic methanogens from a different genus, *Methanobrevibacter*. In phylogenetic analyses of 16S rRNA genes, the *Methanocorpusculum* endosymbiont of *T. finlayi* n. sp. grouped with sequences from Methanomicrobia, including the endosymbiont of an earlier isolate of the same species, ‘*Trimyema* sp.,’ which was sampled approximately 22 years earlier, at a distant (∼400 km) geographical location. Identification of the same endosymbiont species in the two separate isolates of *T. finlayi* n. sp. provides evidence for spatial and temporal stability of the *Methanocorpusculum–T. finlayi* n. sp. endosymbiosis. *T. finlayi* n. sp. and *T. compressum* provide an example of two closely related anaerobic ciliates that have endosymbionts from different methanogen genera, suggesting that the endosymbionts have not co-speciated with their hosts.

## Introduction

Known species of the genus *Trimyema* (class: Plagiopylea, phylum: Ciliophora) are all anaerobic and inhabit diverse environments including freshwater, marine and hypersaline sediments, sewage tanks and hydrothermal vents (Baumgartner et al., 2002; Esteban and Finlay, 2004; Shinzato et al., 2007; Cho et al., 2008). During adaptation to their anaerobic lifestyle, the mitochondria of these ciliates have evolved into hydrogenosomes, mitochondrial homologs that produce H_2_, which is consumed by endosymbiotic methanogenic Archaea (phylum: Euryarchaeota) that live inside the ciliate cells (Augustin et al., 1987; Wagener and Pfennig, 1987; Zwart et al., 1988; Finlay et al., 1993; Lynn, 2008).

Like *Trimyema*, some other microbial eukaryotes can have methanogenic endosymbionts, but they are particularly common in anaerobic ciliates (van Bruggen et al., 1983, 1985; Broers et al., 1990; Finlay et al., 1994; Fenchel and Finlay, 1995). Except for in a handful of cases (Embley et al., 1992a,b; Finlay et al., 1993; Shinzato et al., 2007), the identity of the endosymbiont species has not been reliably established using such methods as species-specific *in situ* probing. Phylogenetic analyses have provided evidence that methanogenic endosymbionts of some ciliates do not evolve in parallel with their hosts and in some cases have been replaced by a new methanogen species (Finlay et al., 1993; van Hoek et al., 2000a). This indicates that the association between methanogenic endosymbionts and their hosts is not entirely stable, and it is possible that a single host species could contain different endosymbionts in specific habitats and at specific times (Embley and Finlay, 1994).

Balanced against the idea that methanogenic endosymbionts are not retained over longer evolutionary time periods, there is evidence from some anaerobic ciliates that their methanogenic endosymbionts are transmitted vertically, and therefore are retained over the evolutionary short-term. For example, the endosymbionts of the ciliate *Plagiopyla frontata* divide in synchrony with their host, which ensures that each daughter host cell receives a number of endosymbionts similar to the number that the mother cell had before division (Fenchel and Finlay, 1991). Likewise, the methanogenic endosymbionts in the ciliate *Metopus palaeformis* were shown to divide at a rate that would ensure a stable population size from one generation of the host to the next (Finlay and Fenchel, 1992). These examples suggest that at least in some anaerobic ciliates, methanogenic endosymbionts have adapted to being vertically transmitted and are not continually replaced by a new methanogen species between host generations. Resampling of endosymbionts from the same host species, isolated at different times and locations, would provide a test of these ideas, and would help us to understand the extent to which these endosymbionts have been retained during the evolutionary history of their hosts.

In 1993, Finlay and colleagues isolated a species of *Trimyema* that was referred to as ‘*Trimyema* sp.’ in several subsequent publications (Embley and Finlay, 1993, 1994; Embley et al., 1995, 2003; Fenchel and Finlay, 1995; Embley, 2006). ‘*Trimyema* sp.’ was described as sharing some morphological similarities to the species *Trimyema compressum* but some distinctions were also highlighted: ‘*Trimyema* sp.’ had fewer somatic kineties than *T. compressum* and both species differed in the structure of their brosse and in their oral infraciliature (Finlay et al., 1993). In the present study, ‘*Trimyema* sp.’ was re-isolated and cultured, identified based on morphometric and molecular data, and demonstrated to be closely related to, but distinct from, *T. compressum*. This new isolate represents a new taxonomic species, which here we describe as *Trimyema finlayi* n. sp. The species of endosymbiotic methanogen in *T. finlayi* was identified by sequencing its 16S rRNA gene, and validated using fluorescent *in situ* hybridization (FISH). A phylogenetic approach was used to investigate the relationship of *T. finlayi* to other ciliates, as well as the relationship of its endosymbiotic methanogen to the methanogenic endosymbiont of *T*. *compressum* and to other methanogenic Archaea. Comparison of the endosymbiont 16S rRNA gene sequences isolated from two closely-related species of ciliates (*T. finlayi* n. sp. and *T. compressum*), as well as those from two isolates of the same species (*T. finlayi* n. sp. and ‘*Trimyema* sp.’) sampled 22 years, and over 400 km apart, provide new insights into spatial and temporal stability of endosymbiosis between anaerobic ciliates and methanogenic Archaea.

## Materials and Methods

### Isolation and Culture of Organisms

Sediment was collected from a freshwater pond located at the East Stoke Fen Nature Reserve (50.679159, -2.191654), close to Wareham, Dorset (United Kingdom), on the floodplain of the river Frome. These samples were collected in April 2013, at which time the depth of the pond did not exceed 1 m. The collected sediment samples were transferred to glass hypo-vials, to which Soil Extract with added Salts (SES) medium was added, prepared according to instructions available from Culture Collection of Algae and Protozoa (CCAP)^1^. Approximately 5 mg of crushed dried cereal leaves and one wheat grain were added to each culture to encourage growth of the naturally existing prokaryotic flora, providing food for the ciliates. The hypo-vials were sealed and their headspace flushed with nitrogen gas for 3 min to remove oxygen, maintaining anoxic conditions within the vials. These enrichment cultures were left to grow for 2 weeks until species of anaerobic ciliates could be microscopically observed in aliquots removed from the cultures. Mono-ciliate cultures were obtained by transferring individual cells to hypo-vials of fresh anoxic culture medium using a glass micropipette. Subculturing was performed monthly by dividing the cultures and then adding fresh media, cereal leaves and wheat grains. All cultures were continually incubated at 18°C.

### DIC Microscopy of Ciliate Cells and F420-Autofluorescence Imaging of Methanogenic Endosymbionts

Living or fixed (4% paraformaldehyde) ciliate cells were imaged using an Olympus BH-2 light microscope and photographed with a Micropublisher 3.3 RTV mounted camera (QImaging). Cell measurements were taken from the images using QCapture Pro software (QImaging). The same microscope and camera was used to detect F420 auto-fluorescence emitted by endosymbiotic methanogens whilst illuminated with UV light (Doddema and Vogels, 1978). To be imaged using this method cells of ciliates were fixed in 4% paraformaldehyde and transferred to a Isopore^TM^ polycarbonate membrane filter (Merck-Millipore), mounted between a microscope slide and cover slip using FF immersion oil (Cargille). Silver carbonate staining of cells was performed as described by Fernández-Galiano (1994).

### DNA Amplification and Sequencing

Polymerase chain reaction was used to amplify the 18S rRNA gene from ciliate cells using KOD Hot Start DNA Polymerase (Merck-Millipore) with the manufacturer’s standard protocol. Five cells were isolated by micropipette, washed three times in sterile PBS, and then transferred to an unsealed microcentrifuge tube, which was then dried at 80°C for 30 min inside a tissue culture hood. This provided the DNA template for the PCR reaction, to which 50 μl of PCR reaction mixture was added. Forward (5′-AYCTGGTTGATYYTGCCAG) and reverse (5′-TGATCCATCTGCAGGTTCACCT) primers (Embley et al., 1992b) were used in an initial PCR reaction to amplify an 1767 base pair fragment of the eukaryotic 18S rRNA gene. The product of this reaction was purified using a QIAquick PCR Purification Kit (QIAGEN) and used as the DNA template of secondary, semi-nested, PCR reactions. One of the semi-nested reactions used the forward primer from the first reaction with the reverse primer EK-1269R (5′-AAGAACGGCCATGCACCAC) (López-García et al., 2001), and the other semi-nested reaction used the forward primer EK-555F (5′-AGTCTGGTGCCAGCAGCCGC) (López-García et al., 2001) and the reverse primer from the first reaction. The same PCR methods were used to amplify the 16S rRNA gene of the *T. finlayi* endosymbiotic methanogen, except the forward primer 340F (5′-CCCTAYGGGGYGCASCAG) (Gantner et al., 2011) and the reverse primer 1100A (5′-TGGGTCTCGCTCGTTG) (Embley et al., 1992b) were used, without a secondary semi-nested reaction.

Thermal cycling conditions used in all PCR reactions were the same as those described by Embley et al. (1992b), except with the addition of an initial heating step at 95°C for 2 min, which was required for the activation of the KOD polymerase. The products of these two semi-nested reactions were purified from a 1% agarose gel using a QIAquick Gel Extraction Kit (QIAGEN), ligated into pJET 1.2 plasmids and cloned using a CloneJET PCR Cloning Kit (Life Technologies) in DH5α cells. Plasmids were purified from overnight cultures using a QIAprep Spin Miniprep Kit (QIAGEN) and five clones for each PCR product were Sanger sequenced in both directions by GATC Biotech using plasmid-specific sequencing primers provided in the cloning kit. Sequencing reads were trimmed and assembled into a complete sequence using the program Sequencher 5.4.6 (Gene Codes Corporation).

### Sequence and Phylogenetic Analysis

For ciliate 18S and methanogen 16S rRNA gene phylogenies, sequences obtained in the present study were aligned with sequences downloaded from GenBank, using the program MUSCLE 3.8.31 (Edgar, 2004). Conserved sites within each dataset were selected and concatenated with the program Gblocks 0.91b (Castresana, 2000). The program jModelTest 2.1.10 (Darriba et al., 2012) selected GTR+Γ+I as the best-fitting model for both alignment datasets. Maximum likelihood analysis was performed with the program RAxML 8.2.4 (Stamatakis, 2014) and statistical support for internal nodes was assessed with 1000 bootstrap replicates. Bayesian analysis was performed using the program MrBayes 3.2.2 (Ronquist and Huelsenbeck, 2003). Two sets of four MCMC chains ran for 1,000,000 generations and were sampled every 100 generations, after which 25% of samples were discarded as burn-in and the standard deviation of split frequencies was below 0.01.

### Fluorescence *in Situ* Hybridization (FISH)

The endosymbiotic methanogen of *T. finlayi* was identified by FISH using the *Methanocorpusculum* oligonucleotide probe, SYM5 (5′-CTGCATCGACAGGCACT) (Finlay et al., 1993), dual labeled with 6-Fam and the positive-control Archaea-specific oligonucleotide probe, ARCH915 (5′-GTGCTCCCCCGCCAATTCCT) (Stahl and Amann, 1991), dual-labeled with Cy3. Both probes were synthesized by biomers.net. Cells were isolated from culture using a micropipette, fixed in 4% paraformaldehyde at 4°C and transferred to poly-L-lysine coated slides. Sample dehydration, probe hybridization and washing were the same as described in Daims et al. (2005). Dried, hybridized samples were mounted on glass cover slides using ProLong Diamond antifade mountant. Z-sections were imaged using a confocal microscope (A1R, Nikon) with a 63x/1.4 objective lens. Vertical z-stacks were deconvolved using Huygens deconvolution software (Scientific Volume Imaging B.V.) with empirically measured point spread functions extracted from images of 0.1 μm TetraSpeck^TM^ Microspheres (Thermo Fisher). Maximum intensity Z-projection images were reconstructed using Fiji (Schindelin et al., 2012).

### Transmission Electron Microscopy

Samples were prepared for transmission electron microscopy (TEM) by centrifuging 200 ml of ciliate cultures at 1500 ×*g* for 45 min. Supernatant was then carefully removed to leave the pellets intact, which were transferred to microcentrifuge tubes. Cells were fixed in 2.5% glutaraldehyde in 0.15 M HEPES-buffer at 4°C. The remaining sample preparation, including post-fixation and embedding, and also imaging of the samples, was performed by Benoît Zuber and Beat Haenni, Microscopy Imaging Center (MIC), Institute of Anatomy, University of Bern, Switzerland, using methods that have been described previously (Tschanz et al., 2003).

## Results

### Morphology of *Trimyema finlayi* n. sp.

**FIGURE 1:**
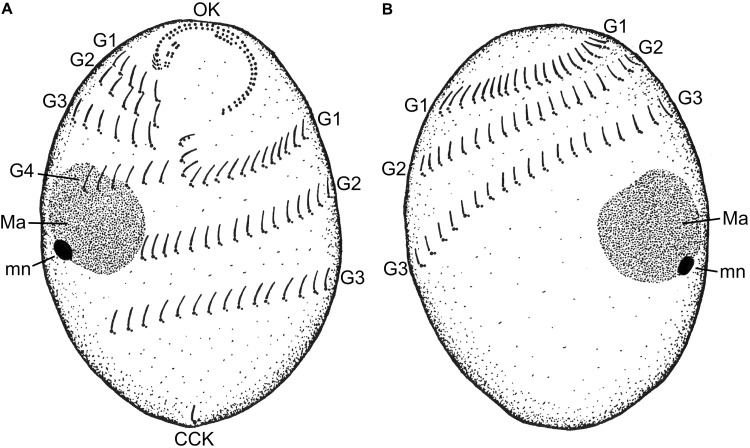
A schematic drawing of the **(A)** ventral and **(B)** dorsal sides of a *Trimyema finlayi* n. sp. cell. CCK, caudal cilium kinety; G1, first ciliary girdle; G2, second ciliary girdle; G3, third ciliary girdle; G4, fourth ciliary girdle; Ma, macronucleus; mn, micronucleus; OK, oral kineties.

**FIGURE 2:**
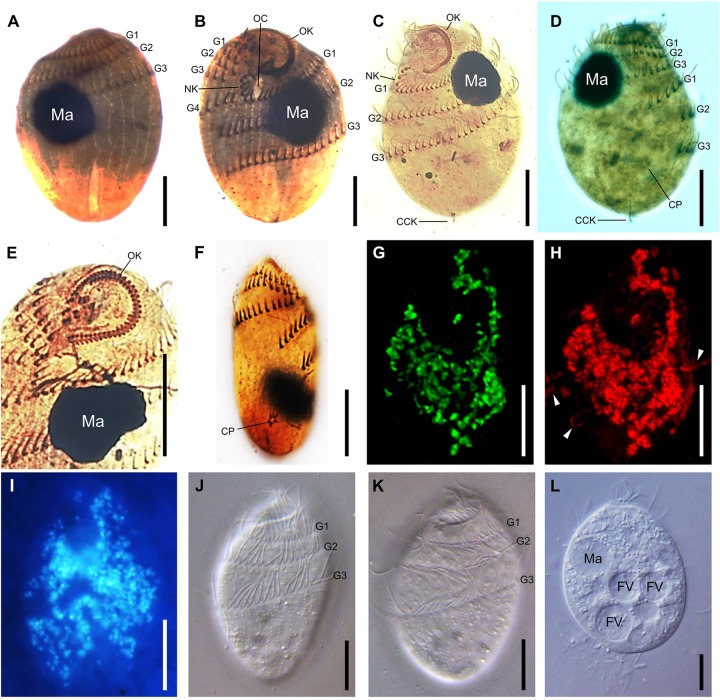
Microscopic imaging of *T. finlayi* n. sp. whole cells. DIC images of silver carbonate impregnated cells, **(A,B)** and **(C,D)** show two sides of the same cells, **(E)** squashed cell showing oral kineties, **(F)** squashed cell showing cytoproct. **(G,H)** Maximum intensity projection of a Z-stack of confocal images across a single *T. finlayi* cell double-labeled with two FISH probes. **(G)** *Methanocorpusculum*-specific probe (SYM5) dual-labeled with 6-FAM. **(H)** Archaea-specific probe (ARCH915) dual-labeled with Cy3, white arrows indicate extracellular Archaea that were not labeled by the probe SYM5 **(G)**. **(I)** F420 auto-fluorescence. **(J–L)** *In vivo* DIC images. CCK, caudal cilium kinety; CP, cytoproct; FV, food vacuole; G1, first ciliary girdle; G2, second ciliary girdle; G3, third ciliary girdle; G4, fourth ciliary girdle; MA, macronucleus; NK, N-kineties; OC, oral cavity; OK, oral kineties. Scale bars = 10 μm.

**FIGURE 3:**
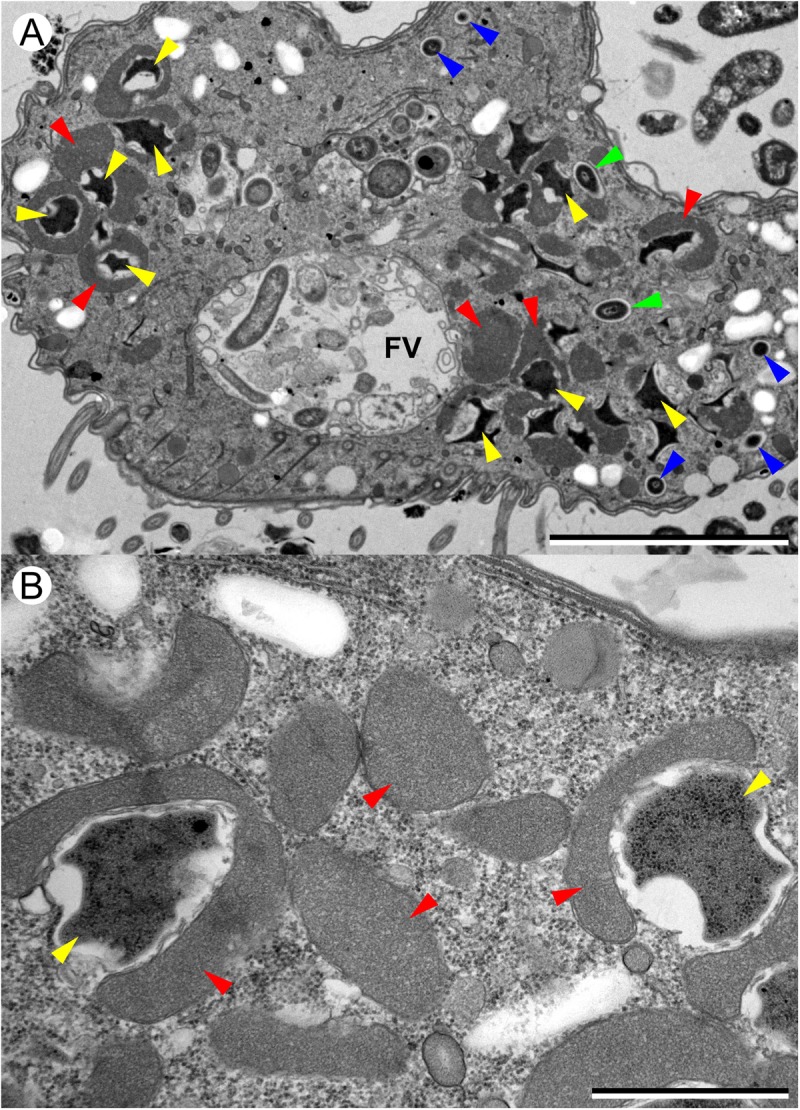
Transmission electron microscopy (TEM) images of *T. finlayi* n. sp. showing polymorphic methanogenic endosymbionts and hydrogenosomes (red arrowheads). Disc-shaped (blue arrowheads) and stellate form (yellow arrowheads) morphotypes are shown, as well as intermediate stages (green arrowheads). FV, food vacuole. Scale bars **(A)** = 5 μm, **(B)** = 1 μm.

The cell shape of *T. finlayi* is a fusiform ellipsoid, tapering toward both the anterior and posterior ends (**Figure 1**). The cell body measured *in vivo* from 60 cells had a length of 27.7–39.9 μm and a width of 17.6–26.6 μm (**Table 1**). *T. finlayi* cells have a single macronucleus, which is positioned off-center from the vertical axis and toward the anterior end of the cell (**Figures 2A–E,L**). The macronucleus is strongly stained by the silver carbonate method (**Figures 2A–F**) and is therefore easy to visualize. The small micronucleus was observed in close proximity to the macronucleus (**Figure 1**) and could not be easily resolved in most of the images from stained specimens. Each cell had 34–45 somatic kineties, organized in longitudinal rows that create the appearance of four ciliary girdles, spiraling obliquely around the cell surface toward the posterior end (**Table 1** and **Figures 2A–D,J,K**). The cell has a single caudal cilium (**Figures 2C,D**), close to which is positioned the cytoproct in the most posterior third of the cell surface (**Figures 2D,F**). The oral cavity is located close to the n-kinety (**Figures 2B,C**). The endosymbiotic methanogens within the ciliate cell appear to form clusters with hydrogenosomes and are distributed throughout the cytoplasm (**Figures 2G–I, 3**).

**Table 1:** Morphometric data characterizing *Trimyema finlayi.*

Characteristics	Method	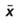	*M*	*SD*	*SE*	CV	Min	Max	*n*
Body length (μm)	IV	34.2	34.0	2.9	0.4	0.1	27.7	39.9	60
Body length (μm)	FF	35.2	35.2	3.6	0.6	0.1	29.3	43.4	37
Body width (μm)	IV	22.1	22.1	2.1	0.3	0.1	17.6	26.6	60
Body width (μm)	FF	25.5	25.4	3.6	0.6	0.1	19.1	34.6	37
Macronuclei number	SC	1.0	1.0	0.0	0.0	0.0	1.0	1.0	15
Oral ciliary rows, number	SC	3.0	3.0	0.0	0.0	0.0	3.0	3.0	15
Kinetids in oral ciliary row	SC	44.3	45.0	2.1	1.2	0.0	42.0	46.0	3
Ciliary girdles on cell body	SC	4.0	4.0	0.0	0.0	0.0	4.0	4.0	15
First ciliary girdle, number of kinetids	SC	39.3	39.0	2.7	0.7	0.1	34.0	43.0	15
Second ciliary girdle, number of kinetids	SC	42.6	43.0	2.2	0.6	0.1	39.0	45.0	15
Third ciliary girdle, number of kinetids	SC	41.3	41.0	1.1	0.3	0.0	40.0	43.0	15
Fourth ciliary girdle, number of kinetids	SC	5.7	6.0	0.5	0.1	0.1	5.0	6.0	15
Number of N kinetids	SC	3.2	3.0	0.4	0.1	0.1	3.0	4.0	15
Caudal cilia number	FF	1.0	1.0	0.0	0.0	0.0	1.0	1.0	37


Holotype and paratypes: A permanent preparation with silver-impregnated specimens has been deposited in the Natural History Museum, London (United Kingdom) (accession: NHMUK 2018.1.30.1) and the species is registered with ZooBank (lsid:zoobank.org:pub:12910A6B-D834-4314-8812-0039F87D6DD2).

Type locality: East Stoke Fen Nature Reserve, East Stoke, Wareham, Dorset (United Kingdom) (50.679159, -2.191654).

Etymology: *finlayi*, dedicated to Professor Bland J. Finlay, in recognition of his many contributions to understanding the ecology of anaerobic ciliates and their endosymbionts, and his impact on the field of microbial ecology more generally.

### Phylogenetic Relationships of *Trimyema finlayi* n. sp.

**FIGURE 4:**
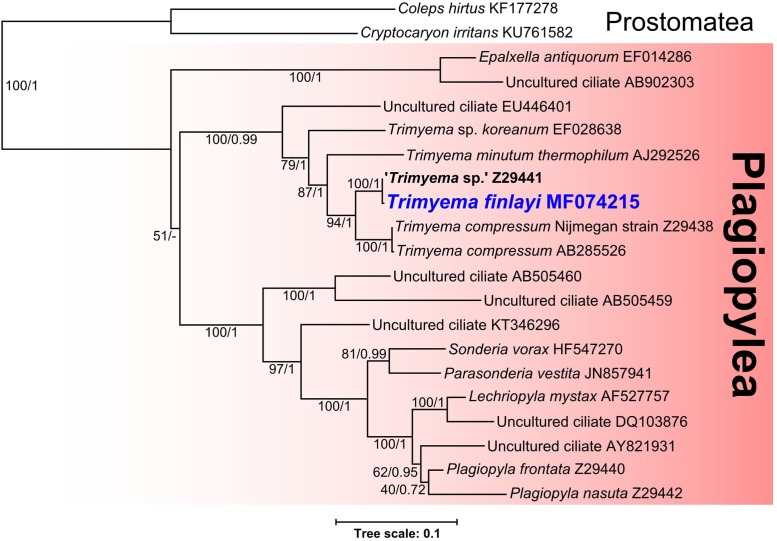
Bayesian phylogeny inferred from 1640 nucleotide alignment of 18S rRNA genes of Plagiopylea species using the GTR+Γ+I model. Support values represent maximum likelihood bootstrap support/Bayesian posterior probabilities. Scale bar represents the number of substitutions per site.

Phylogenetic analysis of the 18S rRNA gene sequence for *T. finlayi* (accession number: MF074215) (**Figure 4**) suggests that it is most closely related to ‘*Trimyema* sp.’ (bootstrap support = 100, posterior probability = 1) and comparable sequenced regions of their 18S rRNA genes are 99.6% identical when aligned. These two sequences form a sister group (bootstrap support = 94, posterior probability = 0.96) to a clade containing two sequences from *T. compressum* (bootstrap support = 100, posterior probability = 1) and they are also more closely related to other *Trimyema* sequences than other species of Plagiopylea (**Figure 4**).

### Identification and Morphology of Endosymbiotic Methanogens Living in *Trimyema finlayi* n. sp.

F420 autofluorescence (**Figure 2I**) indicated the presence of methanogens within cells of *T. finlayi*. In order to identify the species of these methanogens, a 16S rRNA gene was sequenced from isolated ciliate cells, which was 99% identical to sequences from several species of the genus *Methanocorpusculum* in GenBank, including *Methanocorpusculum parvum* and *Methanocorpusculum aggregans*. In FISH experiments, Archaea labeled with a *Methanocorpusculum*-specific oligonucleotide probe, SYM5, were localized inside *T. finlayi* cells, but not outside (**Figure 2G**). A positive-control Archaea-specific oligonucleotide probe, ARCH915, bound to the endosymbiotic methanogens, as well as extracellular Archaea present in the sample (**Figure 2H**).

Transmission electron microscopy images indicate that the endosymbiotic methanogens in *T. finlayi* are polymorphic, consisting of two main morphotypes: cells of the first morphotype appear smaller and more round (**Figure 3**, blue arrowheads) and are have previously been described as ‘disc-shaped’ (Finlay et al., 1993). Cells of the second morphotype are larger and more irregular in shape (**Figure 3**, yellow arrowheads), with their cell walls more invaginated; cells of this morphotype have previously been described as ‘stellate forms’ (Finlay et al., 1993). The endosymbiont cells of the stellate-form morphotype are also typically closely associated with hydrogenosomes (**Figure 3**, red arrowheads) and in some cases appear almost completely encapsulated by them. In addition, there appear to be some intermediate forms between these two morphotypes (**Figure 3**, green arrowheads), suggesting that the endosymbionts undergo transformation from one form to the other, as observed by Finlay et al. (1993).

Several findings support the idea that the endosymbionts are two morphotypes of the same species (Finlay et al., 1993): firstly, based on TEM images (**Figure 3**), in the case of all morphotypes, the center of the methanogen is electron-dense and is surrounded by a less electron-dense outline that varies in thickness. Secondly, the endosymbionts appear similar when labeled with different FISH-probes (**Figures 2G,H**), as well as when imaged based on their F420-autofluorsence (**Figure 2I**). Additionally, each of these images looks like those of earlier isolates (i.e., ‘*Trimyema* sp.’), which were made using similar methods (Finlay et al., 1993). Finally, the Archaea-specific FISH-probe (**Figure 2H**) co-localizes with the *Methanocorpusculum*-specific FISH-probe (**Figure 2G**), suggesting that all of the archaeal cells within *T. finlayi* are the same species of the genus *Methanocorpusculum*.

### Phylogenetic Relationships of Endosymbiotic Methanogens from *Trimyema* Species and Their Free-Living Relatives

**FIGURE 5:**
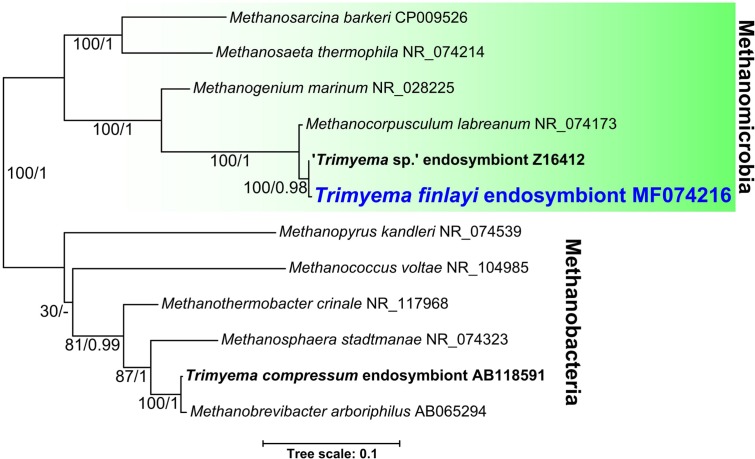
Bayesian phylogeny inferred from a 1372 nucleotide alignment of methanogenic Archaea 16S rRNA genes using the GTR+Γ+I model. Support values represent maximum likelihood bootstrap support/Bayesian posterior probabilities. Scale bar represents the number of substitutions per site.

In order to investigate the relationship between the endosymbiotic methanogens of *Trimyema* species and other methanogenic Archaea, the 16S rRNA gene of the endosymbionts from *T. finlayi* (accession number: MF074216) was sequenced (from hand-picked and washed ciliate cells) and analyzed phylogenetically, together with the 16S rRNA genes of other methanogens (**Figure 5**). The endosymbiotic methanogens of *T. finlayi* and ‘*Trimyema* sp.’ (Finlay et al., 1993) grouped together (bootstrap support = 100, posterior probability = 0.98), and they both formed a clade with the free-living methanogen species *Methanocorpusculum labreanum* (bootstrap support = 100, posterior probability = 1), within a larger clade that includes sequences from other species in the order Methanomicrobiales (bootstrap support = 100, posterior probability = 1). Identification of only a single 16S rRNA gene sequence from *T. finlayi* (this study) and ‘*Trimyema* sp.’ (Finlay et al., 1993) isolates, provides further support for the hypothesis that the two types of archaeal cells, observed inside the *T. finlayi* cytosol in TEM images (**Figure 3**), are two morphotypes of a single archaeal species. The endosymbiotic methanogen of the ciliate *T. compressum* did not group with the endosymbionts of ciliates from the same genus, ‘*Trimyema* sp.’ and *T. finlayi*, as was suggested previously (Shinzato and Kamagata, 2010), and is consistent with the hypothesis that the endosymbiosis has been established more than once during the evolution of the *Trimyema* lineage. Instead the endosymbiont of *T. compressum* forms a clade with species in the order Methanobacteriales (bootstrap support = 100, posterior probability = 1) and is most closely related to the free-living methanogen *Methanobrevibacter arboriphilus* (bootstrap support = 100, posterior probability = 1). This is consistent with a previous study that identified the methanogenic endosymbiont of *T. compressum* as a member of the Methanobacteria genus *Methanobrevibacter* by using FISH with a species-specific probe (Shinzato et al., 2007).

## Discussion

Morphological descriptions of *T. compressum* differ between publications (Augustin et al., 1987; Wagener and Pfennig, 1987; Serrano et al., 1988) and a general consensus seems to be lacking. Therefore morphological parameters of *T. finlayi* were compared to three previously published descriptions of *T. compressum* (Augustin et al., 1987; Wagener and Pfennig, 1987; Serrano et al., 1988) as well as a previously published partial description of ‘*Trimyema* sp.’ (Finlay et al., 1993) (**Table 2**). The measured mean length of *T. finlayi* (34.7 μm) was lower than the mean length of *T. compressum* (39.05–65.9 μm), based on all three descriptions and falls within the range that was specified for ‘*Trimyema* sp.’ (30–50 μm). The range in number of somatic kineties recorded for ‘*Trimyema* sp.’ (37–40) falls within the range measured for *T. finlayi* (34–45), whereas the range in number of somatic kineties for *T. compressum* is systematically higher (50–60) (Augustin et al., 1987; Serrano et al., 1988).

**Table 2:** Comparison of morphometric data collected in separate studies for species of *Trimyema.*

Species	Mean length (μm)	Mean width (μm)	*n*	Longitudinal (somatic) kineties	Shape of methanogens
*Trimyema finlayi*^1^	34.2	22.1	97	34–45	Polymorphic
‘*Trimyema* sp.’^2^	n/a	n/a	n/a	37–40	Polymorphic
*Trimyema compressum*^3^	39.05	22.3	20	50–60	Rod
*Trimyema compressum*^4^	65.9	54.6	48	50–60	Rod
*Trimyema compressum*^5^	40	25	n/a	n/a	Rod


Phylogenetic analysis of the 18S rRNA genes sequenced from these ciliates (**Figure 4**) suggests that *T. finlayi* and ‘*Trimyema* sp.’ form a clade (bootstrap support = 100, posterior probability = 1) that is a sister group to sequences from *T. compressum* (bootstrap support = 94, posterior probability = 1). The small number of nucleotide differences (6 substitutions; 1479 compared bases) between the 18S rRNA gene sequences from *T. finlayi* and ‘*Trimyema* sp.’ could be a consequence of inter-strain differences, due to them being isolated at different times and locations (South and North of England, respectively). Alternatively, since the ‘*Trimyema* sp.’ sequence (accession number: Z29441.1) contains 12 ambiguous bases, this suggests that the overall quality of the sequence is relatively low, and therefore these differences between the two sequences could be the result of sequencing errors. A comparable number of nucleotide differences (2 substitutions; 1616 compared bases) is also observed between the 18S rRNA gene sequences from two isolates of *T. compressum* (accession numbers: AB285526.1 and Z29438.1). Some of the sequences included in the phylogenetic analysis shown in **Figure 4** were obtained from environmental sequencing studies that have sampled a vast variety of geographical locations (Šlapeta et al., 2005; Zuendorf et al., 2006; Alexander et al., 2009; Takishita et al., 2010; Matsunaga et al., 2014; Pasulka et al., 2016). The ciliates from these studies are uncultured and 18S rRNA gene sequences provide the only evidence for their existence, which indicates that there is species-level diversity within the class Plagiopylea that remains uncharacterized.

Studies listed in **Table 2** (Augustin et al., 1987; Wagener and Pfennig, 1987; Serrano et al., 1988), as well as a more recent study (Shinzato et al., 2007), describe *T. compressum* as having rod-shaped endosymbiotic methanogens, whereas fluorescence (**Figures 2G–I**) and TEM images (**Figure 3**) show that *T. finlayi* has irregularly-shaped endosymbiotic methanogens. Furthermore, the general morphology, cellular distribution and overall appearance of the endosymbionts from *T. finlayi*, as well as their associations with hydrogenosomes, appear to be very similar to previously published TEM images of ‘*Trimyema* sp.’ (Finlay et al., 1993). The 16S rRNA genes of the endosymbiotic methanogens in *T. finlayi* and ‘*Trimyema* sp.’ are 99.5% identical (2 substitutions; 443 compared bases) and phylogenetic analysis with other methanogen sequences (**Figure 5**) suggests that they are closely related to each other (bootstrap support = 100, posterior probability = 0.98) and belong to the genus *Methanocorpusculum*. In contrast, the endosymbiont of *T. compressum* is related to members of the genus *Methanobrevibacter* (**Figure 5**), which supports the findings of a previous study (Shinzato et al., 2007).

In addition to containing an endosymbiotic methanogen, *T. compressum* was previously shown to also contain a bacterial endosymbiont, closely related to the species *Petrimonas sulfuriphila* (Shinzato et al., 2007). We found no evidence however, from FISH experiments using a Bacteria-specific probe, to suggest that *T. finlayi* has a bacterial endosymbiont.

Our findings provide robust morphological and molecular evidence to suggest that *T. finlayi* and ‘*Trimyema* sp.’ are two isolates of the same species, which from this point forward should be referred to as *T. finlayi*. We have also shown that this species is distinct from but closely related to *T. compressum*.

Previous studies have provided evidence that methanogenic endosymbionts of anaerobic ciliates do not co-speciate over the long-term with their hosts, suggesting that the endosymbionts of some anaerobic ciliates have occasionally been replaced by another species (Finlay et al., 1993; van Hoek et al., 2000b). Thus, closely-related hosts may have methanogen endosymbionts from different genera and *vice versa* (Embley and Finlay, 1994). Our results further support a lack of long-term co-speciation between host and symbionts in the *Trimyema* lineage – while the hosts *T. compressum* and *T. finlayi* (formerly ‘*Trimyema* sp.’) clearly belong to the same genus, the endosymbiotic methanogens of these two species are not closely related (**Figure 5**) (Shinzato and Kamagata, 2010). In the case of *T. finlayi*, however, there does appear to be stability of these associations in the evolutionary short-term (i.e., spatially and temporally isolated samples of the same species). Thus, *T. finlayi* (formerly ‘*Trimyema* sp.’) has now been isolated on two different occasions from distant geographical locations as part of separate studies, several years apart, and both isolates contain closely-related endosymbionts belonging to the genus *Methanocorpusculum* (**Figure 5**). *T. finlayi* was initially isolated from Priest Pot, a pond in Cumbria, northern England, United Kingdom (Finlay et al., 1993), and in the present study from a pond in East Stoke Fen, Dorset, southern England, United Kingdom. These two sites are separated by over 400 km and were sampled approximately 22 years apart. The finding that at least some anaerobic ciliates retain their endosymbiotic methanogens over the evolutionary short-term indicates that the symbiotic consortium is not entirely transient.

The observed *Methanocorpusculum* endosymbionts in *T. finlayi* are polymorphic (Finlay et al., 1993), and differed from the typical coccoid morphology of some of their closest known free-living relatives (Anderson et al., 2009). Some of the endosymbiont cells formed close associations with the ciliates hydrogenosomes, which is likely to be an adaptation to their endosymbiotic lifestyle, allowing them to uptake H_2_ with increased efficiency (Finlay et al., 1993). Similar observations have been made in the ciliate *Metopus contortus*, which also has polymorphic endosymbionts of the genus *Methanocorpusculum*, and also seem to undergo a morphological transformation (Embley et al., 1992a), suggesting that species of the genus *Methanocorpusculum* might share homologous adaptations that facilitate their endosymbiotic lifestyle.

The endosymbionts of *T. finlayi* appear to transform their morphology, presumably to form closer associations with hydrogenosomes, which suggests that these two organisms have evolved a relatively stable association. In contrast, although the endosymbionts of *T. compressum* can also be closely associated with hydrogenosomes (Shinzato et al., 2007), they typically appear rod-shaped and therefore resemble other free-living methanogen species of the same genus (*Methanobrevibacter*) (Wagener and Pfennig, 1987; Goosen et al., 1990). There are also reported cases where methanogenic endosymbionts were lost from *T. compressum* in laboratory cultures (Wagener and Pfennig, 1987; Wagener et al., 1990; Holler and Pfennig, 1991). In some of these cases the ciliates re-incorporated the endosymbionts when they were co-incubated with a pre-grown methanogen culture (Wagener et al., 1990). These observations suggest that the endosymbiont of *T. compressum* may be less adapted to an endosymbiotic lifestyle, and provides evidence that the association between these species is less evolutionarily stable in comparison to the corresponding symbiosis in *T. finlayi*. Alternatively, the capacity to lose and subsequently re-establish endosymbionts within its cells could be a mechanism used by *T. compressum* to adapt to a changing environment.

Additional sampling, together with reliable *in situ* identification, of endosymbiotic methanogens living within other congeneric ciliate species, would provide further insight into the extent, or lack of, co-speciation between host and endosymbiont. Sequencing the genomes of the methanogenic endosymbionts from both *T. finlayi* and *T. compressum*, and comparing them with the genomes of their close free-living relatives, could also provide molecular insights into the relative stability of these associations, by identifying general or species-specific patterns of gene loss or gain that have allowed certain methanogens to become endosymbionts.

## Author Contributions

WL carried out field work, molecular lab work, microscopy and bioinformatic data analysis, and drafted the manuscript. KS carried out molecular lab work, TME coordinated and helped to design aspects of the study, and GE conceived the study, identified the species and carried out field and lab work.

## Conflict of Interest Statement

The authors declare that the research was conducted in the absence of any commercial or financial relationships that could be construed as a potential conflict of interest.

## Footnotes

1 https://www.ccap.ac.uk/media/documents/SES.pdf
